# Sicca manifestations and lymphoproliferation in hepatitis C virus: effects of direct acting antiviral therapy on dryness and B-cell activity compared to Sjögren’s disease

**DOI:** 10.1186/s13075-025-03605-9

**Published:** 2025-07-07

**Authors:** Amina Maher, Mohamed Tharwat Hegazy, Tareq M. Algarf, Manar A. Abdul-Aziz, Luca Quartuccio, Naguib Zoheir, Salvatore De Vita, Gaafar Ragab

**Affiliations:** 1 Internal Medicine Department, Rheumatology and Clinical Immunology Unit, Faculty of Medicine, Cairo University, Cairo, Egypt; 2 Department of Otorhinolaryngology, Faculty of Medicine, Cairo University, Cairo, Egypt; 3 Oral Pathology Department, Faculty of Oral & Dental Medicine, Cairo University, Cairo, Egypt; 4 Clinic of Rheumatology, Department of Medical Area (DAME), University Hospital “Santa Maria della Misericordia”, University of Udine, Udine, Italy; 5 Clinical and Chemical Pathology Department, Faculty of Medicine, Cairo University, Cairo, Egypt

**Keywords:** Hepatitis C virus, Sjögren’s disease, Direct-acting antiviral drugs, B-cell proliferation, Sicca manifestations

## Abstract

**Objectives:**

Hepatitis C virus (HCV) can be associated with sicca manifestations. To study the effect of direct-acting antivirals (DAAs) on sicca manifestations in HCV-infected patients and the difference between those patients and others with HCV without dryness & Sjögren’s disease (SjD).

**Methods:**

We studied 60 patients in 3 groups: Group 1 (20 HCV + sicca), group 2 (20 HCV without sicca), and group 3 (20 SjD). Groups 1 and 2 received DAAs according to the Egyptian Ministry of Health protocols and were evaluated before and after treatment. Group 3 was evaluated once. Our study evaluated the patients by both subjective and objective methods.

**Results:**

All HCV cases had sustained viral response (SVR). Comparing the characteristics of groups 1 (before treatment) & 3: Group 1 had a higher frequency of RF, cryoglobulins, and polyclonal-hypergammaglobulinemia (*P*-values 0.021, 0.003, and ˂0.001 respectively). Group 3 had higher scores of VAS dry eye, VAS dry mouth, VAS fatigue, and VAS pain than group 1 (*P*-values ˂0.001 in all). Group 3 also had a higher frequency of Anti-Ro and Anti-La (*P*-values < 0.001). Group-1 before DAAs treatment had higher markers denoting B-cell hyperactivity [higher Rheumatoid factor (RF), cryoglobulins, and beta2-microglobulins (β2M)] compared to group-2 which improved markedly after SVR. This supports that group 1 is further ahead in the direction of lymphoproliferation. Group 1 patients after SVR showed marked improvement in VAS dry eye, VAS dry mouth, VAS fatigue, VAS pain, ESSPRI, and ESSDAI (*P*-values ˂0.003, ˂0.002, ˂0.016, ˂0.001, ˂0.002, and ˂0.014 respectively). There was a significant improvement in RF, and serum β2M levels (after SVR), (*P*-values ˂0.013, and 0.001 respectively). Group 1 is further ahead in the direction of lymphoproliferation than group 2 with higher statistically significant serum β2M and polyclonal serum protein electrophoresis (*P*-values 0.006 and 0.047 respectively).

**Conclusion:**

HCV patients with sicca manifestations treated by DAAs showed significant clinical and immunological improvements. The difference between group 1 (before and after SVR) and group 3 supports the notion that they are two different entities, with different characteristic features. Sicca manifestations improved after the eradication of HCV.

**Supplementary Information:**

The online version contains supplementary material available at 10.1186/s13075-025-03605-9.

## Introduction

Hepatitis C virus (HCV) is linked to many autoimmune features and extra-hepatic manifestations, impacting morbidity, mortality, and medical costs [1]. Sicca symptoms, including dry eyes and dry mouth, are reported in 10–30% of cases [2]. Sjögren’s disease (SjD) and HCV share B-cell hyperactivity characteristics. Also, HCV can infect salivary and lacrimal gland tissues, leading to lymphocytic infiltration. HCV patients with sicca symptoms are typically older and have a higher incidence of extraglandular manifestations compared with SjD patients. As for their serological findings, they are more likely to have cryoglobulinemia, negative Ro/La antibodies, and hypocomplementemia [3].

Approximately 50% of HCV-infected patients exhibit chronic focal sialadenitis resembling SjD, while signs of mild inflammatory infiltration are present in a greater proportion of patients [4]. However, sicca symptoms appear to be less frequent and milder in HCV-infected patients compared to SjD patients, raising questions about HCV as a possible triggering agent for SjD [5]. Both share common features such as cryoglobulinemia and hypocomplementemia which are commonly found in HCV-infected patients and are considered markers or predictors for the development of some extra-hepatic manifestations [6] and associated autoimmune or lymphoproliferative disorders [7].

Chronic HCV infection doubles the risk of developing non-Hodgkin lymphoma (NHL), which increases to approximately 35-fold in patients with symptomatic HCV-associated mixed cryoglobulinemia [8, 9]. Similarly, SjD patients have a significantly higher risk of NHL compared to the general population [10]. Key predictors for NHL development include low complement levels, cryoglobulinemia, lymphadenopathy, ectopic germinal center-like structures, permanent parotid gland swelling, skin vasculitis [10, 11], and elevated levels of beta2-microglobulin (β2M) as well as free light chains of immunoglobulins [12].

Our objectives were to study the effects of the new Direct-Acting Antivirals (DAAs) and consequently viral clearance on sicca manifestations in HCV-infected patients (group 1), their effect on B cell proliferation, and whether or not this could decrease the risk of development of NHL. We also aimed to explore the degree of lymphoproliferation in HCV patients with Sicca in comparison to those with HCV without dryness as well as Sjögren’s disease. We also aimed to describe the characteristics of dryness in HCV cases and cases with Sjögren and also the changes that occur after HCV clearance.

To the best of our knowledge, this is the first study to explore the effect of anti-HCV treatment on sicca manifestations in HCV patients by both subjective and objective methods.

## Patients and methods

### Study design

We performed an observational analytical study between 2019 and 2022 which included 60 patients divided into three equal groups: HCV-RNA positive patients with sicca manifestations [Oral dryness and eye dryness by subjective and objective methods adopted from the 2016 ACR-EULAR Classification Criteria for Sjögren’s Disease [7] (group 1), HCV-RNA positive patients without Sicca manifestations (group 2), and Sjögren’s disease patients (group 3) [7]. All patients were from the same socioeconomic background.

### Exclusion criteria

1. Patients who did not fulfill any of the criteria for the 3 groups.
2. Patients with other autoimmune diseases or receiving drugs influencing dryness as anti-histamines, anti-depressants, diuretics, B-blockers, etc.

### Ethical component

All procedures performed in the study were in accordance with the ethical standards of the National Research Committee and the 1964 Helsinki Declaration and its later amendments and approved by the Research Ethics Committee, Faculty of Medicine, Cairo University (REC code: D-28-19). A written informed consent was obtained from all participating patients.

Our comparative study was not planned as a clinical trial. Our data were derived from HCV patients who received anti-HCV medications according to the protocols approved and endorsed by the Egyptian Ministry of Health during the national campaign to eradicate HCV.

### Data collection

Patients were recruited from Kasr Al-Ainy outpatient clinics.

Groups 1 and 2 received treatment with DAAs and were assessed at baseline and after 6 months from the end of therapy with DAAs. Group 3 was assessed similarly only once.

### Assessment included

A. Full Clinical evaluation by history, systemic examination, and assessment of sicca manifestations using visual analogue scores (VAS dry eye, VAS dry mouth, and VAS fatigue) as well as EULAR Sjogren’s Syndrome Patient Reported Index (ESSPRI) [13] and single items and EULAR Sjögren’s Syndrome Disease Activity Index (ESSDAI) and single domains [14].
B. Objective tests for dryness:
  Schirmer’s test [15](abnormal ≤ 5 mm/5min),
  Ocular staining by fluorescein stain [16] (positive ≥ 5).
  Unstimulated salivary flow (USSF) rates assessment [17] (abnormal ≤ 0.1mL/min).
C. Laboratory investigations including CBC, liver and kidney function tests, serum cryoglobulins, rheumatoid factor (RF) by Nephelometry, antinuclear antibodies (ANA) by IF with titer and pattern, anti-Ro and anti-La, complement 3 (C3), complement 4 (C4) and serum protein electrophoresis (SPEP), IgG, serum β2M as well as HCV viral load (quantitative PCR for HCV- RNA).
D. Minor salivary gland (MSG) biopsies [18] for those patients after explaining the procedure, formalin-fixed: 4 lobules were blindly evaluated by an experienced pathologist for assessment of focus score [19] (the number of mononuclear cell infiltrates containing at least 50 inflammatory cells in a 4 mm2 glandular section). Which was calculated by using a computer image analyzer, the surface area of the salivary gland was measured, lymphocytes were counted and the focus score was calculated according to the following equation,
  $${\rm Focus\, score} =\:\frac{number\:of\:lymphocytic\:aggregates\:(\ge\:50)}{surface\:area\:of\:salivary\:gland}\:X\:4$$
E. Abdominal ultrasonography.

### Evaluation of the clinical and immunological response of HCV patients in groups 1 and 2 was done 6 months after finishing anti-HCV treatment

1. The improvement of subjective manifestations using VAS for dry eye, dry mouth, fatigue, and pain is defined as a 30% or greater reduction of VAS [20].
2. Improvement of objective tests of dryness: (Change before and after therapy: number (%) of patients in which the test became normal and was initially abnormal): Schirmer’s test, Ocular staining, and USSF test.
3. ESSPRI is defined as satisfactory symptoms state if < 5 and unsatisfactory symptoms state ≥ 5, therapeutic response is considered as an improvement of at least 1 point [13].
4. ESSDAI is defined as mild disease < 5, moderately active disease 5–13, and severely active disease if ≥ 14 (change before and after therapy: variation in minimal clinically important improvement (MCII) defined as a decrease of at least 3 points [14].
5. Sjögren’s Tool for Assessing Response (STAR): Also, the response was evaluated using STAR [21]. The result of ≥ 5 points was considered as STAR responder.

## Results

### Baseline characteristics of group 1, group 2 and group 3 (Tables 1 and 2) and (Fig. 1)

**Table 1:** Baseline characteristics of group 1, group 2 and group 3:

	Group 1			Group 2			Group 3		
	Mean	SD	Median	Mean	SD	Median	Mean	SD	Median
**Age in years**	46.55	10.26	50.00	37.75	12.46	36.50	41.05	10.83	40.00
**Duration of Sicca in years**	7.05	4.17	6.50	--------	-------	-------	7.60	2.30	8.00
**VAS dry eye**	5.05	2.164	5.00	-------	-------	-------	8.70	1.750	10.00
**VAS dry mouth**	5.10	2.553	5.00	-------	-------	-------	9.45	1.234	10.00
**VAS fatigue**	5.40	2.604	5.00	2.95	3.591	0.00	9.15	1.599	10.00
**VAS pain**	4.075	2.337	5.00	0.65	0.933	0.00	8.75	1.209	9.00
**ESSPRI**	4.90	2.049	5.00	1.10	1.294	0.00	9.05	1.317	10.00
**ESSDAI**	0.55	0.759	0.00	0.40	0.598	0.00	2.95	3.284	2.00
**Schirmer’s Test**	8.50	9.417	4.50	---------	---------	-------	7.30	8.291	3.00
**USSF test**	1.285	0.7727	1.000	---------	--------	-------	0.860	0.7315	1.000
**RF (0–14 IU/ml)**	34.450	44.7552	15.300	15.685	13.0067	10.500	16.750	21.1390	9.750
**β2M (0.6–2.29 mcg/ml)**	2.19	0.55	2.40	1.80	0.63	1.60	2.44	1.15	2.40
**IgG titre**	1,539.40	538.068	1,525.00	1,564.55	1,029.558	1,405.00	1,548.40	828.321	1,263.50

VAS: Visual analogue scale, ESSDAI: EULAR Sjögren’s syndrome disease activity index, ESSPRI: EULAR Sjogren’s syndrome Patient Reported Index, RF: Rheumatoid factor, USSF: Unstimulated salivary flow, β2M: beta2-microglobulin

**Fig. 1:**
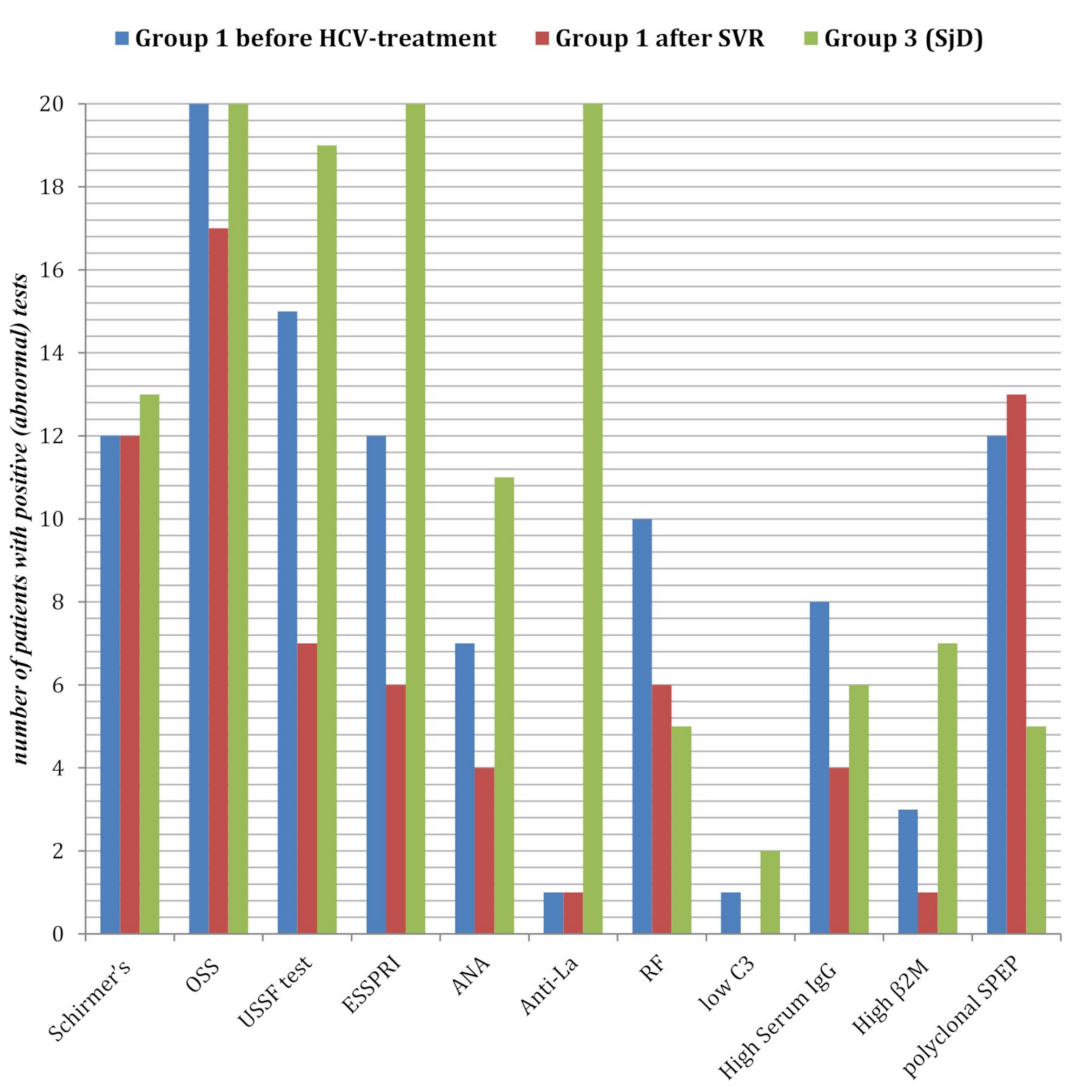
Comparison between the characteristics of group 1 (before and after HCV treatment by DAAs) and group 3 OSS: Ocular staining surface, USSF: Unstimulated salivary flow, ANA: Anti-nuclear antibodies, RF: Rheumatoid factor, C3: Complement 3, β2M: beta2-microglobulin, SPEP: serum protein electrophoresis, DAAs: Direct antiviral Agents,

**Table 2:** Baseline characteristics of group 1, group 2 and group 3:

		Group 1 *N*= (20)	Group 2 *N*= (20)	Group 3 *N*= (20)
**Sex**	**Male**	9	14	1
	**Female**	11	6	19
**DM**	**Yes**	0	0	0
	**No**	20	20	20
**HTN**	**Yes**	0	0	3
	**No**	20	20	17
**HCV**		Yes	Yes	No
**Child classification**	**A**	19	20	---------
	**B**	1	0	---------
	**C**	0	0	---------
**Schirmer’s test**	**Positive**	12	0	13
	**Negative**	8	20	7
**OSS**	**Positive**	20	0	20
	**Negative**	0	20	0
**USSF**	**Positive**	15	0	19
	**Negative**	5	20	1
**ANA**	**Positive**	7	6	11
	**Negative**	13	14	9
**Anti Ro**	**Positive**	0	0	16
	**Negative**	20	19	4
	**Borderline**	0	1	0
**Anti La**	**Positive**	1	1	13
	**Negative**	18	18	7
	**Borderline**	1	1	0
**C3**	**Normal**	19	19	18
	**Low**	1	1	2
**C4**	**Normal**	19	18	20
	**High**	1	0	0
	**Low**	0	2	0
**Cryoglobulins**	**Positive**	8	3	0
	**Negative**	12	17	20
**SPEP**	**Normal**	8	9	15
	**Polyclonal**	12	11	5
	**Monoclonal**	0	0	0

OSS: Ocular staining surface, RF: Rheumatoid factor, USSF: Unstimulated salivary flow, ANA: Anti-nuclear antibodies, C3: Complement 3, C4: Complement 4, SPEP: serum protein electrophoresis

Group 1 included 20 patients, their ESSPRI showed that 12 patients (60%) had unsatisfactory symptoms. All 20 patients (100%) in group 1 had mild or absent disease activity according to ESSDAI.

In group 2, a total of 20 patients were included. During the assessment, all patients were evaluated for extra-hepatic and autoimmune manifestations, and none of them exhibited any such manifestations.

Group 3 included 20 patients, 10 patients (50%) of them had arthralgia of whom only one patient (5%) had arthritis with morning stiffness < 10 min. Also, only one patient (5%) had Raynaud’s phenomenon. Three patients (15%) had bilateral parotid gland enlargement.

All 20 patients (100%) in group 3 had ESSPRI unsatisfactory symptoms (ESSPRI > 5). ESSDAI showed that 16 Patients (80%) had mild disease activity, 3 patients (15%) had moderate disease activity and only one patient (5%) had severe disease activity.

### Post-sustained viral response (SVR) characteristics of group 1 (Tables 3 and 4) and (Fig. 1)

**Table 3:** Comparison of group 1 before and after DAAs

Group 1	Pretreatment					Post treatment					*P* value
	Mean	SD	Median	Minimum	Maximum	Mean	SD	Median	Minimum	Maximum	
**VAS dry eye**	5.05	2.164	5.00	2	9	3.70	2.886	3.00	0	10	0.003
**VAS dry mouth**	5.10	2.553	5.00	2	10	2.95	3.187	2.00	0	10	0.002
**VAS fatigue**	5.40	2.604	5.00	2	9	3.70	3.404	2.00	0	10	0.016
**VAS pain**	4.75	2.337	5.00	1	9	2.80	2.505	2.00	0	9	0.000
**ESSPRI**	4.90	2.049	5.00	1	9	3.45	2.685	3.50	0	10	0.002
**ESSDAI**	0.55	0.759	0.00	0	2	0.25	0.550	0.00	0	2	0.014
**Schirmer’s Test**	8.50	9.417	4.50	0	30	10.10	10.305	4.50	1	35	0.106
**USSF test**	1.285	0.7727	1.000	0	3	1.870	0.7263	2.000	1	3	0.010
**RF (0–14 IU/ml)**	34.450	44.7552	15.300	7.3	193	18.54	14.333	10.25	8	46	0.013
**β2M (0.6–2.29 mcg/ml)**	2.19	0.55	2.40	1.60	3.20	1.66	0.52	1.60	0.80	2.59	0.001
**IgG titre**	1,539.40	538.068	1,525.00	724	3,050	1,476.40	344.526	1,513.00	780	2,390	0.218

VAS: Visual analogue scale, ESSDAI: EULAR Sjögren’s syndrome disease activity index, ESSPRI: EULAR Sjogren’s syndrome Patient Reported Index, RF: Rheumatoid factor, USSF: Unstimulated salivary flow, β2M: beta2-microglobulin, DAAs: Direct antiviral Agents

**Table 4:** Characteristics of group 1 after DAAs

Group 1		Pre-treatment *N* = 20	Post- treatment *N* = 20
**Schirmer’s test**	Positive	12	12
	Negative	8	8
**OSS**	Positive	20	17
	Negative	0	3
**USSF**	Positive	15	7
	Negative	5	13
**ANA**	Positive	7	4
	Negative	13	16
**Anti Ro**	Positive	0	0
	Negative	20	20
	Borderline	0	0
**Anti La**	Positive	1	1
	Negative	18	19
	Borderline	1	0
**C3**	Normal	19	20
	Low	1	0
**C4**	Normal	19	18
	High	1	2
	Low	0	0
**Cryoglobulins**	Positive	8	0
	Negative	12	20
**SPEP**	Normal	8	7
	Polyclonal	12	13
	Monoclonal	0	0

OSS: Ocular staining surface, USSF: Unstimulated salivary flow, ANA: Anti-nuclear antibodies, C3: Complement 3, C4: Complement 4, SPEP: serum protein electrophoresis, DAAs: Direct antiviral Agents

VAS dry eye response showed that 10 patients (50%) improved. VAS dry mouth response showed that 13 patients (65%) improved. VAS fatigue response showed that 13 patients (65%) improved. VAS pain response showed that 15 patients (75%) improved. ESSPRI response after SVR was 14 patients (70%) showing improvement and 6 patients (30%) with no improvement.

Schirmer’s test response after SVR was two patients had the test becoming normal, though initially abnormal (14.3%). In 10 patients (71.4%) the test remained abnormal and in two other patients (14.3%) the test became abnormal although it was initially normal.

USSF test response after SVR detected 9 patients having the test becoming normal whereas it was initially abnormal (56.3%). In 6 patients (37.5%) the test remained abnormal and in one patient (6.3%) the test became abnormal despite being initially normal.

As for the improvement of patients’ sicca symptoms and signs, after achieving SVR, using ESSPRI was that 14 patients (70%) improved while 6 patients did not. The results of STAR showed that 11 patients (55%) improved while 9 patients (45%) did not.

IgG response after SVR showed that 8 patients (40%) had a 10% decrease in its level while 12 patients (60%) did not.

#### 3 patients had minor salivary gland (MSG) biopsy before and after treatment

One patient had diffuse sialadenitis that remained the same after SVR (had improvement according to Sjögren’s Tool for Assessing Response (STAR). One patient had focal sialadenitis with a focus score < 2 that remained the same after SVR (i.e. improvement according to STAR). Another patient had focal sialadenitis with a focus score < 2 that turned to diffuse sialadenitis (no improvement according to STAR). One patient had a biopsy with no yield for MSG tissue.

### Group 1 comparison between findings before and after treatment (Table 3)

There was a statistically significant improvement in VAS dry eye, VAS dry mouth, VAS fatigue, and VAS pain figures in group 1 patients after SVR when compared to those before starting anti-viral treatment (*P*-values 0.003, 0.002, 0.016, and 0.000 respectively). There was a high statistically significant improvement in ESSPRI and ESSDAI in group 1 patients after SVR (*P*-values 0.002 and 0.014 respectively).

There was no statistically significant improvement in Schirmer’s test (*P*-value 0.106). There was a statistically significant improvement in the USSF test (*P*-value 0.010). There was a statistically significant improvement in RF and serum β2M in group 1 patients after SVR (*P* values 0.013 and 0.001 respectively). There was no statistically significant improvement in IgG (*P*-value 0.218).

### Group 2 comparison between findings before and after treatment (supplementary table 1)

There was a reduction in the number of positive cryoglobulins (15% before treatment to 0% after treatment). There was no statistically significant improvement in RF, IgG, IgM, and IgA after SVR (*P*-values 0.293, 0.794, 0.097, and 0.538 respectively). There was no change in the number of ANA-positive patients (30% remained the same).

### Comparison between group 1 & group 2 before HCV treatment (Supplementary Table 2a)

Group 2 had distinctive features.

There was a statistically significant difference in SPEP, like the presence of polyclonal gammopathy in group 1 (*P*-value 0.047).

There was a statistically significant difference in serum β2M, being higher in group 1 (*P*-value 0.006).

There was no statistically significant difference in RF and IgG (*P*-values < 0.191 and < 0.501 respectively).

There was no statistically significant difference in the levels of ANA, Anti-Ro, Anti-La, C3, and C4 (*P*-values 0.736, 1, 1, 1, and 0.487, respectively).

(These findings support the concept that group 1 is further ahead in the direction of lymphoproliferation).

### Comparison between group 1 & group 2 after treatment (Supplementary Table 2b)

There was no statistically significant difference in ANA, RF, and RF response between both groups (*P*-values 0.716, 0.716, and 0.411 respectively).

There was no statistically significant difference in IgG and IgG decrease by 10% between the two groups (*P*-values 1.0 and 0.501 respectively).

### Comparison between group 1 before DAAs and group 3 patients (Table 5)/ different characteristics of dryness

**Table 5:** Comparison between group 1 (before treatment) and group-3

	Group 1 (Pre-treatment)					Group 3					*P* value	
	Mean	SD	Median	Minimum	Maximum	Mean	SD	Median	Minimum	Maximum		
**VAS dry eye**	5.05	2.164	5.00	2	9	8.70	1.750	10.00	5	10	0.000	
**VAS dry mouth**	5.10	2.553	5.00	2	10	9.45	1.234	10.00	6	10	0.000	
**VAS fatigue**	5.40	2.604	5.00	2	9	9.15	1.599	10.00	5	10	0.000	
**VAS pain**	4.75	2.337	5.00	1	9	8.75	1.209	9.00	7	10	0.000	
**ESSPRI**	4.90	2.049	5.00	1	9	9.05	1.317	10.00	7	10	0.000	
**ESSDAI**	0.55	0.759	0.00	0	2	2.95	3.284	2.00	0	13	0.004	
**Schirmer’s Test**	8.50	9.417	4.50	0	30	7.30	8.291	3.00	0	25	0.644	
**USSF test**	1.285	0.7727	1.000	0	3	0.860	0.7315	1.000	0	3	0.137	
**RF titre (0–14)**	34.450	44.7552	15.300	7.3	193	16.750	21.1390	9.750	6	96	0.021	
**β2M**	2.19	0.55	2.40	1.60	3.20	2.44	1.15	2.40	0.60	5.60	0.547	
**IgG titre**	1,539.40	538.068	1,525.00	724	3,050	1,548.40	828.321	1,263.50	858	4,330	0.543	

VAS: Visual analogue scale, ESSDAI: EULAR Sjögren’s syndrome disease activity index, ESSPRI: EULAR Sjogren’s syndrome Patient Reported Index, RF: Rheumatoid factor, USSF: Unstimulated salivary flow, β2M: beta2-microglobulin

There was a high statistically significant difference in VAS dry eye score, VAS dry mouth score, VAS fatigue, and VAS pain between the two groups being higher in group 3 patients (*P*-values < 0.001 in all). There was a high statistically significant difference in ESSPRI and ESSDAI between the two groups where more unsatisfactory symptoms response and more severe disease were found in group 3 (*P*-values (< 0.001) and 0.004). There was no statistically significant difference in Schirmer’s test and USSF test results between the two groups (*P*-values 0.659 and 0.0.157 respectively). There was a high statistically significant difference in Anti-Ro and Anti-La antibodies between the two groups being more positive in group 3 (*P*-values < 0.001 in both). There was a statistically significant difference in RF level between the two groups being higher in group 1 (*P*-value 0.021). There was a statistically significant difference in serum cryoglobulins levels of the patients between the two groups being more positive in group 1 (*P*-value 0.003). There was a high statistically significant difference in SPEP percentage of the patients between the two groups being more abnormal in the form of polyclonal gammopathy in group 1 (*P*-value < 0.001). There was no statistically significant difference in β2M levels of the patients between the two groups (*P*-value 0.547). There was no statistically significant difference in ANA, C3, C4 level, and IgG findings between the two groups (*P*-values 0.204, 1.00, 1.00, and 0.547 respectively).

## Discussion

To the best of our knowledge, this is the first study to explore the effect of anti-HCV treatment (DAAs) and viral eradication on sicca manifestations in HCV patients both subjectively and objectively. It differs from previous studies in that they checked for HCV infection among patients who were previously diagnosed with SjD [6, 22–24].

In our study, comparing sicca manifestations in group 1 before and after SVR showed improvement in patients’ response according to STAR whereas 11 patients (55%) showed improvement after viral eradication.

This agrees with Doffoel-Hantz et al., 2005 [25], who prospectively studied 12 patients with HCV-associated sicca manifestations treated with interferon or interferon/ribavirin dual therapy for their HCV in the pre-DAAs era. They reported that the sicca manifestations improved upon treatment in 5/12 cases, and after stopping treatment three (3/12) cases maintained their improvement. In four out of five cases, the patients were treated with dual therapy (Interferon/ribavirin). Their assessment, however of sicca manifestations was made by questioning only and they didn’t apply VAS or any other objective assessment.

In our study, group 3 patients had higher statistically significant VAS dry eye score, VAS dry mouth score, VAS fatigue, and VAS pain than group 1 (*P*-values < 0.001 in all). Also, group 3 patients had a higher frequency of Anti-Ro (80% vs. 0%) and Anti-La (65% vs. 5%) antibodies (*P*-values < 0.001 in both) in comparison with group 1. In group 3, serum β2M was high in 35% of patients (mean 2.44 ± 1.15 SD).

On the other hand, group 1 patients, compared with group 3 had a statistically higher frequency of RF (50% vs. 25%), serum cryoglobulins (40% vs. 0%), and polyclonal hypergammaglobulinemia (60% vs. 25%) with (*P*-values 0.021, 0.003 and 0.00 respectively).

In their extensive work, Brito-Zerón, et al., 2015 [24], studied the prevalence of HCV among 783 patients with SjD and reported 105 patients positive for HCV. Patients with both HCV and sicca manifestations (compared with SjD without HCV) had a higher frequency of RF (56% vs. 40%, *P*-value 0.004), serum monoclonal gammopathy (47% vs. 17%, *P*-value < 0.001), and cryoglobulinemia (61% vs. 7%, *P*-value < 0.001); a lower frequency of ANA (76% vs. 84%, *P*-value 0.05), anti-Ro (15% vs. 43%, *P*-value < 0.001), and anti-La (17% vs. 30%, *P*-value < 0.001).

Ceribelli, et al., 2008 [23], studied nine cases out of 305 SjD patients who were concomitantly affected by SjD and HCV infection. Patients with HCV and sicca manifestations had a higher mean age (74 years vs. 61, *P*-value 0.01), and they showed a higher prevalence of circulating cryoglobulins (44.4% vs. 13.3%, *P*-value 0.0372), with no clinical manifestations of cryoglobulinemia during the period of follow-up. They detected positive ANA in (100%) of their nine patients, isolated anti-Ro in (66.6%) and anti-Ro associated with anti-La in (33.3%). Their SjD patients as a whole had isolated anti-Ro (49.8%) or associated with anti-La (46.5%) of patients, i.e. the presence of HCV seemed to have altered the pattern of autoimmune serological positivity.

In Ramos-Casals, 2005 [6], a multicenter study defined the clinical and immunological pattern of 137 patients with HCV infection-associated and sicca manifestations. HCV-infected individuals with sicca symptoms compared to SjD patients were older at diagnosis. They also had a higher rate of positive RF (50% vs. 38%, *P*-value 0.009), cryoglobulinemia (50% vs. 9%, *P*-value < 0.00), and hypocomplementemia (51% vs. 12%, *P*-value 0.001). Also, HCV patients had lower rates of ANA (65% vs. 74%), anti-Ro (21% vs. 40%, *P* < 0.000), and anti-La (16% vs. 26%, *P*-value 0.014) antibodies. These results seem to be echoing our findings.

Even in an earlier work, Ramos-Casals, et al., 2001 [22] studied the clinical and immunologic description of 35 HCV patients with sicca manifestations in comparison with SjD patients. They reported that HCV patients had a higher mean age (65.9 ± 1.3 years versus 61.5 ± 1.4 years, *P*-value 0.04), Moreover, those patients with HCV showed a higher prevalence of cryoglobulinemia (60% versus 10%, *P*- value 0.001), and hypocomplementemia (60% versus 8%, *P*- value 0.001), and a lower prevalence of anti-Ro/SS-A (17% versus 38%, *P*- value 0.03) when compared with SjD patients.

In our study, there was a statistically significant improvement in RF and serum β2M in group 1 patients after SVR as compared to those before starting DAAs (P-values 0.013 and 0.001 respectively).

There was no statistically significant improvement in IgG in group 1 patients after SVR as compared to those before starting DAAs (*P*-value 0.218).

The comparison of group 1 and group 2 showed that the presence of sicca manifestations in HCV patients points to a disease that is more severe and has moved forward in the direction of lymphoproliferation (before HCV treatment, evident by the finding that group 1 patients had a significantly higher level of β2M than group 2 with *P*- value 0.006).

Pertovaara and his team (2001) [26], reported that patients with SjD who subsequently developed lymphoma had higher baseline serum β2M levels than the others. Thus, the possibility of developing malignant lymphoproliferation should be considered, especially in patients with raised baseline serum β2M concentrations. In the study of Gottenberg and his group (2013) [12], high levels of β2M and free light chains of immunoglobulins were associated with high disease activity and B-cell stimulation (serum polyclonal hypergammaglobulinemia, raised levels of β2m and B-cell activating factor levels) as well as serum cryoglobulins, RF, low C3/C4 complement factors and serum monoclonal immunoglobulins.

All these observations weigh heavily in favor of recommending observing the level of β2M as it may predict the evolution of lymphoproliferation.

In recapitulation, group 3 patients had higher statistically significant VAS dry eye score, VAS dry mouth score, VAS fatigue, and VAS pain figures (*P*- value 0.001 in all) when compared with group 1. Also, group 3 patients had a higher frequency of anti-Ro (80% vs. 0%) and anti-La (65% vs. 5%) antibodies (*P*-values < 0.001 in both) in comparison with group 1. Also in group 3, Serum β2M was high in 35% of patients (mean 2.44 ± 1.15 SD).

On the other hand, group 1 patients had a statistically higher frequency of RF (50% vs. 25%), serum cryoglobulins (40% vs. 0%), and polyclonal hypergammaglobulinemia (60% vs. 25%) with *P*-values 0.021, 0.003 and 0.00 respectively than group 3.

So, we think group 1 patients (HCV with sicca manifestations had higher markers denoting B-cell proliferation (higher RF, cryoglobulins, and β2M) that markedly improved after viral eradication. We recommend longer follow-up periods for these patients. We also suggest that HCV patients with sicca should be prioritized for early treatment in case of limited drug availability to lower the risk of lymphoproliferation.

The difference between group 1 (before and after SVR) and group 3 supports the notion that they are two different entities, with different characteristic features. Also, the improvement of Sicca manifestations after HCV eradication calls for considering the revision of the 2016 ACR-EULAR Classification Criteria for primary Sjögren’s Syndrome, which states that active HCV infection by polymerase chain reaction is an exclusion criterion for diagnosis of Primary Sjögren. Based on our findings, we suggest that even treated HCV infection should be excluded from the Classification Criteria for primary Sjögren’s disease.

### This study has some limitations including

1. The relatively small number of the studied patients in each group.
2. The short follow-up period, which was cut short by the outbreak of the COVID-19 pandemic. However, our results reported changes after HCV eradication even within a short time span.
3. The small number of MSG biopsies in HCV patients was a challenging issue as many patients refused to perform biopsies based on their mild sicca manifestations in group 1 and no sicca manifestations in group 2. The small number of MSG biopsies could be increased in the future by the use of Salivary gland ultrasound (SGUS). SGUS stands a chance to play an important role in SjD clinical practice and research as a guide for core needle biopsy (CNB), a feasible and accurate technique that recent studies proved to be safer than the current open surgical biopsy [27, 28].

## Conclusion

Treatment of patients with HCV and sicca manifestations by DAAs is associated with significant clinical and immunological improvement. HCV patients with sicca manifestations have higher markers of B-cell proliferation (higher RF, cryoglobulins, and β2M) that improved markedly after DAAs. We emphasize the need for a longer follow-up observation for these patients. We also recommend their prioritization for early treatment. This could lower the risk of progressive lymphoproliferation.

We also suggest the addition of treated HCV infection to the list of exclusions of the classification criteria for Sjögren’s disease.

## Electronic supplementary material

Below is the link to the electronic supplementary material.

Supplementary Material 1

## Data Availability

No datasets were generated or analysed during the current study.

## Abbreviations

ACR: American College of Rheumatology
ALT: Alanine transaminase
ANA: Anti-nuclear antibodies
AST: Aspartate aminotransferase
C3: Complement 3
C4: Complement 4
CBC: Complete blood count
CNB: Core needle biopsy
CRP: C-reactive protein
DAAs: Direct antiviral Agents
ESSDAI: EULAR Sjögren’s syndrome disease activity index
ESSPRI: EULAR Sjogren’s syndrome Patient Reported Index
EULAR: European Alliance of Associations for Rheumatology
HCV: Hepatitis C virus
Ig: Immunoglobulin
MSG: Minor salivary gland
NHL: Non-Hodgkin lymphoma
OSS: Ocular staining surface
PCR: Polymerase chain reaction
RF: Rheumatoid factor
RNA: Ribonucleic acid
SD: Standard deviation
SGUS: Salivary gland ultrasound
SjD: Sjögren’s disease
SPEP: Serum protein electrophoresis
STAR: Sjögren’s Tool for Assessing Response
SVR: Sustained viral response
USSF: Unstimulated salivary flow
USWSF: Unstimulated whole salivary flow
VAS: Visual analogue scale
β2M: Beta2-microglobulin
