# Supplementary material for: Sicca manifestations and lymphoproliferation in hepatitis C virus: effects of direct acting antiviral therapy on dryness and B-cell activity compared to Sjögren’s disease
Source: Arthritis Res Ther. 2025 Jul 7;27:139. doi: 10.1186/s13075-025-03605-9 (PMC12232862; doi:10.1186/s13075-025-03605-9)
Supplement: Supplementary file 1 — Supplementary Material 1 [file 13075_2025_3605_MOESM1_ESM.docx]

**Supplementary Table 1: Comparison of group 2 (before and after DAAs)**

| **Group 2** | **Pre-treatment** | | | | | **Post-treatment** | | | | | **P value** |
| --- | --- | --- | --- | --- | --- | --- | --- | --- | --- | --- | --- |
|  | **Mean** | **SD** | **Median** | **Minimum** | **Maximum** | **Mean** | **SD** | **Median** | **Minimum** | **Maximum** |  |
| **VAS fatigue** | 2.95 | 3.591 | 0.00 | 0 | 10 | 1.10 | 2.614 | 0.00 | 0 | 10 | 0.024 |
| **VAS pain** | 0.65 | 0.933 | 0.00 | 0 | 3 | 0.15 | 0.366 | 0.00 | 0 | 1 | 0.014 |
| **ESSPRI** | 1.10 | 1.294 | 0.00 | 0 | 3 | 0.40 | 0.883 | 0.00 | 0 | 3 | 0.019 |
| **ESSDAI** | 0.40 | 0.598 | 0.00 | 0 | 2 | 0.25 | 0.550 | 0.00 | 0 | 2 | 0.257 |
| **RF titre** | 15.685 | 13.0067 | 10.500 | 6 | 47 | 11.93 | 3.278 | 10.00 | 10 | 19 | 0.293 |
| **β2M** | 1.80 | 0.63 | 1.60 | 0.80 | 3.50 | 1.88 | 0.68 | 1.70 | 1.00 | 4.20 | **0.375** |
| **IgG titre** | 1,564.55 | 1,029.558 | 1,405.00 | 775 | 5,710 | 1,615.10 | 984.959 | 1,395.00 | 870 | 5,690 | 0.794 |

**ALT:** Alanine transaminase**, AST:** Aspartate aminotransferase**, ESSDAI:** EULAR Sjögren's syndrome disease activity index, **ESSPRI:** EULAR Sjogren's syndrome Patient Reported Index, **RF:** Rheumatoid factor, **USSF:** Unstimulated salivary flow, **VAS:** Visual analogue scale, **β2M:** beta2-microglobulin, PC: prothrombin concentration, **DAAs:** Direct antiviral Agents

**Supplementary Table 2**

**Supplementary Table 2a: Comparison between group 1 and group 2 before DAAs**

|  | **Group 1** | | | | | **Group 2** | | | | | **P value** |
| --- | --- | --- | --- | --- | --- | --- | --- | --- | --- | --- | --- |
|  | **Mean** | **SD** | **Median** | **Minimum** | **Maximum** | **Mean** | **SD** | **Median** | **Minimum** | **Maximum** |  |
| **RF (0-14)** | 34.450 | 44.7552 | 15.300 | 7.3 | 193 | 15.685 | 13.0067 | 10.500 | 6 | 47 | **0.107** |
| **β2M** | 2.19 | 0.55 | 2.40 | 1.60 | 3.20 | 1.80 | 0.63 | 1.60 | 0.80 | 3.50 | **0.006** |
| **IgG (700-1600)** | 1,539.40 | 538.068 | 1,525.00 | 724 | 3,050 | 1,564.55 | 1,029.558 | 1,405.00 | 775 | 5,710 | **0.449** |

**RF:** Rheumatoid factor, **β2M:** beta2-microglobulin, **Ig:** Immunoglobulin, **DAAs:** Direct antiviral Agents

**Supplementary Table-2b:**

**Comparison between group 1 and group 2 after DAAs**

|  | **Group 1** | | | | | **Group 2** | | | | | **P value** |
| --- | --- | --- | --- | --- | --- | --- | --- | --- | --- | --- | --- |
|  | **Mean** | **SD** | **Median** | **Minimum** | **Maximum** | **Mean** | **SD** | **Median** | **Minimum** | **Maximum** |  |
| **RF (0-14)** | 18.54 | 14.333 | 10.25 | 8 | 46 | 11.93 | 3.278 | 10.00 | 10 | 19 | 0.366 |
| **β2M** | 1.66 | 0.52 | 1.60 | 0.80 | 2.59 | 1.88 | 0.68 | 1.70 | 1.00 | 4.20 | 0.301 |
| **IgG (700-1600)** | 1,476.40 | 344.526 | 1,513.00 | 780 | 2,390 | 1,615.10 | 984.959 | 1,395.00 | 870 | 5,690 | 0.409 |

**RF:** Rheumatoid factor, **β2M:** beta2-microglobulin, **Ig:** Immunoglobulin, **DAAs:** Direct antiviral Agents
